# The *QUEST* for Effective and Equitable Policies to Prevent Non-communicable Diseases: Co-Production Lessons From Stakeholder Workshops

**DOI:** 10.34172/ijhpm.2020.99

**Published:** 2020-06-28

**Authors:** Ffion Lloyd-Williams, Rebecca Masters, Lirije Hyseni, Emily St. Denny, Martin O’Flaherty, Simon Capewell

**Affiliations:** Department of Public Health, Policy and Systems, University of Liverpool, Liverpool, UK.

**Keywords:** Co-Production, Policy, Inequalities, Public Health, NCDs

## Abstract

**Background:** Non-communicable diseases (NCDs) account for some 90% of premature UK deaths, most being preventable. However, the systems driving NCDs are complex. This complexity can make NCD prevention strategies difficult to develop and implement. We therefore aimed to explore with key stakeholders the upstream policies needed to prevent NCDs and related inequalities.

**Methods:** We developed a theory-based co-production process and used a mixed methods approach to engage with policy- and decision-makers from across the United Kingdom in a series of 4 workshops, to better understand and respond to the complex systems in which they act. The first and fourth workshops (London) aimed to better understand the public health policy agenda and effective methods for co-production, communication and dissemination. In workshops 2 and 3 (Liverpool and Glasgow), we used nominal group techniques to identify policy issues and equitable prevention strategies, we prioritised emerging policy options for NCD prevention, using the MoSCoW approach.

**Results:** We engaged with 43 diverse stakeholders. They identified ‘healthy environment’ as an important emerging area. Reducing NCDs and inequalities was identified as important, underpinned by a frustration relating to the evidence/ policy gap. Evidence for NCD risk factor epidemiology was perceived as strong, the evidence underpinning the best NCD prevention policy interventions was considered patchier and more contested around the social, commercial and technological determinants of health. A comprehensive communications strategy was considered essential. The contribution of ‘elite actors’ (ministers, public sector leaders) was seen as key to the success of NCD prevention policies.

**Conclusions:** NCDs are generated by complex adaptive systems. Early engagement of diverse stakeholders in a theory-based co-production process can provide valuable context and relevance. Subsequent partnership-working will then be essential to develop, disseminate and implement the most effective NCD prevention strategies.

## Background

Non-communicable diseases (NCDs) account for some 90% of premature deaths in the United Kingdom,^1^ yet most are preventable. Risk factors for NCDs include poverty, poor diet and obesity, tobacco, alcohol and physical inactivity. The Global Burden of Disease, Injuries and Risk Factors study examined the patterns of poor health in the United Kingdom, and identified that despite universal free access to healthcare, the United Kingdom has some of the poorest health outcomes for NCDs when compared to similar European countries.^2^ Around a quarter of the UK population, some 15 million people live with a chronic condition, many NCD related.^3^NCDs annually cost the United Kingdom over £130Bn, representing an immense burden on the National Health Service, the economy and wider society.^3^

The relationship between NCDs and the wider context of people’s lives is complex. The social determinants of health, such as housing, education, employment and transport all powerfully impact on the health of individuals and their wider communities.^4^ Traditional approaches to prevention have had significant successes in improving public health, such as tobacco control and safer motor vehicles.^5-7^ However, even more effective approaches are required in order to stem the rising tide of chronic diseases.

The key NCD drivers include poor diet, smoking and alcohol; all being non-linear and unpredictable complex adaptive systems characterised by emergence, feedback and adaptation.^8^ This complexity can make it difficult to define and measure the impact of specific policies and interventions.^8^ Effective NCD prevention therefore needs to better understand this complexity, mapping the system’s visible elements, functional interconnections, purpose, paradigms and structures.^9^Only then can one realistically test potential solutions. Policy-makers and other research users likewise operate in complex policy-making systems; while many appreciate the potential benefit of systems thinking, some still desire further evidence of its value to guide actions in the real world.^10^

In 2018, we established the QUEST Research Consortium to help address and shape the prevention agenda for NCDs, to explore what are the upstream policies needed to prevent NCDs and related inequalities? We aimed to help policy-makers better understand and respond to the complex systems in which they act, in order to produce actionable evidence to prevent NCDs and reduce associated inequalities. We focused on advancing actions relating to food, tobacco and alcohol policies, in order to produce compelling evidence to help reduce both premature NCDs and associated inequalities by 33% by 2030, (the WHO Sustainable Development Goal 3.4).^11^

## Methods

Our mixed methods study first identified key policy- and decision-makers from across the United Kingdom, using networking and snowball techniques.^12^ In Workshops 1 and 4, we discussed and learnt about the upcoming NCD Prevention policy agenda at local and UK levels, and how best to influence it. In Workshops 2 and 3, we used nominal group techniques to identify policy issues and the most equitable NCD prevention strategies to help translate compelling evidence into policy,^13^ and prioritised emerging policy options using the MoSCoW prioritisation approach^14^ to identify short, medium and long term priorities for NCD prevention. Workshop 4, our final workshop, also focused on the most effective methods for co-production,^10,12^ communication and dissemination of optimal strategies.

Working with policy-makers, we iteratively developed a co-production process,^15,16^ in order to shape and evaluate emerging policy options. We wanted to identify the most equitable prevention strategies, then help translate this compelling evidence into policy and practice, by integrating diverse stakeholder perspectives, best evidence and a systems approach^5,17^ into our innovative, quantitative policy models.

This process was built on a solid theoretical foundation, one which recognises that the value of co-production between researchers and research users for policy improvement rests not only in the generation of actionable policy-relevant knowledge, but also in the fostering of strong collaborative relationships.^10^ Furthermore, insights from the literature on successful policy-making underscore the importance of dialogue between researchers and policy actors to translate evidence into effective action and help close the evidence-policy ‘gap.’^18^In this case, the different actors are not considered to be from separate and distinct professional ‘communities,’ but rather part of the complex system in which policy-relevant knowledge is generated and organised into action.^19,20^

###  Stakeholder Recruitment

We identified and invited senior decision and policy-makers offering national, regional and local perspectives. Participants were invited via various methods: direct invitations to known topic experts, via colleagues and via snowball sampling.^21^ The final 43 participants included senior decision-makers from a wide variety of organisations, including Public Health England (national and regional level), the National Institute for Health and Care Excellence, UK government, local authorities, academic institutions, research institutions, and a wide range of national and international third sector organisations (Table 1) and disciplines (health and well-being, environment/sustainability, public health intelligence/policy/science/research, heart disease prevention, communications, health economics, population health, childhood health/obesity, nutrition and health, physical activity, and alcohol, drugs and tobacco control). Workshop 1 and 4 participants primarily represented national perspectives (location: London) and Workshop 2 and 3 participants primarily local and regional perspectives (location: Liverpool and Glasgow). The rationale for this approach was to identify priorities for NCD prevention interventions and policies at all government levels and reflect the divergent public health systems any successful NCD-reduction policies will have to appropriate for the devolved governments of the United Kingdom.

**Table 1:** *QUEST* Stakeholder Workshop Attendees

**Stakeholder Workshop 1 (London)**	**Stakeholder Workshop 2 (Liverpool)**
(5 Females/2 males) Chief Economist, large public health body Director of Policy and Global Health Deputy Chief Executive, NGO Policy and Campaigns Manager Analyst, national body Consultant Public Health Adviser Senior Programme Manager, NGO	(9 Females/2 males) Public Health Consultant, LA Specialty Registrar in Public Health Obesity Lead, Regional Body Health and Well-being Programme Lead, NGO Director of Research, large Public Health Organisation Consultant in Public Health Medicine, LA Chief Executive, NGO Director for Health and Well-being, LA Public Health Consultant, National Body Deputy Medical Director Programme Director
**Stakeholder Workshop 3 (Glasgow)**	**Stakeholder Workshop 4 (London)**
(10 Females/3 males) Consultant in Public Health Medicine, LA Chief Executive, NGO Chief Executive, Local Authority Senior Communications and Engagement Officer, Large Public Health Body Director of Public Health, National Body Consultant in Public Health, HB Consultant in Public Health, HB Director of Research, National Body Organisational Lead, LA Public Health Intelligence Advisor Organisational Lead, National Body Public Health Intelligence Principal, LA Economic Adviser, National Body	(5 Females/7 males) International Business Development Director, NGO Senior Policy and Research Executive, Large Public Health Body Charity Chief Executive, NGO Senior Policy and Research Executive, NGO Head of Policy, Large Public Health Body Campaigns and Policy Manager, NGO Charity Chief Executive, NGO Director of Policy and Global Health Charity Deputy Chief Executive Head of Business Development Head of Policy Policy and Public Affairs Officer

Abbreviations: NGO, non-governmental organisation; LA, local authority; HB, health board.

 All participants were informed in advance of the purpose and format of the workshops. Verbal consent for group feedback and discussion to be recorded in note form was obtained. No identifiable individual comments or expressed views were used.

###  Workshop Design and Nominal Group Techniques

We conducted 4 stakeholder workshops with 43 senior public health policy- and decision-makers from around the United Kingdom. The first and fourth workshops (in London) aimed to better understand the public health policy agenda^8^ and develop our shared vision and values, via facilitated roundtable discussions and feedback with senior decision- and policy-makers. At the beginning of each workshop, participants were informed about the purpose and aims of the workshop and project. Participants were encouraged to ask questions to clarify their role, expectations, workshop format and outcomes of their contribution. Workshop 1 specifically explored the upcoming policy agenda and what research and evidence was required by policy-makers to inform change. Workshop 4 also focused on the better understanding of the potential QUEST contribution towards NCD prevention. We aimed to identify the most effective methods for co-production,^15,16^ communication and dissemination and explored how QUEST could have the optimum impact at a national level.

The second and third workshops took place in Liverpool and Glasgow respectively. We utilised nominal group techniques (a structured method for group brainstorming that encourages contributions from everyone. In small groups (n = 3-4 participants per group in QUEST workshops) members begin by writing down their ideas, then select which idea they feel is best. Once team members are ready, everyone presents their favourite idea, and the suggestions are then discussed and prioritized by the entire group).^22^ to develop and prioritise a comprehensive list of possible prevention policies using local, regional and national perspectives.

###  Prioritisation Using the MoSCoW Approach

We identified current and future short, medium and long-term priorities for the next 1, 5 and 10 years using the MoSCoW prioritisation approach.^14^ That is based on 4 categories:

- *Must have: the suggestions are critical to delivery and without these, the action will fail.*
- *Should have: the suggestions are important but are not as time dependent as the suggestions in the ‘must have’ category. *
- *Could have: the suggestions are desirable but not necessary.*
- *Would have: the suggestions are least important to delivery and can be either dropped or incorporated at a later stage. *

Table 2 provides a summary of the workshop aims and activities.

**Table 2:** Summary of Workshop Aims and Activities

**Workshop**	**Aims**	**Activity**
**Workshop 1**	To learn about upcoming policy agenda, and how to influence it To develop QUEST community To develop our shared vision and values To agree principles of collaborative working, based on our policy colleagues’ advice	* Activity: Presentations from policy colleagues (as listed in Table 1) on: * Current and future policy priorities (1, 5, and 10 year) How policy agendas change Spotting windows of opportunity Questions and Answers session Learning Points
**Workshop 2**	Discuss some principles of collaborative working Discuss and learn about the upcoming NCD Prevention policy agenda at local and UK levels, and how best to influence it Build an understanding of QUEST potential contribution to NCD prevention Build a comprehensive list of possible NCD prevention policies	*Activity 1: Presentations from policy colleagues with expertise in obesity, health and well-being, research on:* Current and future prevention policy priorities (1, 5, and 10 years) How policy agendas change Spotting windows of opportunity *Activity 2: Consensus Building Workshop* What are the upstream policies needed to prevent NCD and related inequalities? (Group discussion and Plenary) Brainstorming possible NCD prevention policies; building a longlist; Shortlisting NCD prevention policies
**Workshop 3**	Discuss and learn about the upcoming NCD Prevention policy agenda at local and UK levels, and how best to influence it Build an understanding of QUEST potential contribution to NCD prevention Build a comprehensive list of possible NCD prevention policies	*Activity 1: Presentations from policy colleagues with expertise in public health intelligence, communications, alcohol on:* Current and future prevention policy priorities (1, 5, and 10 years) How policy agendas change Spotting windows of opportunity *Activity 2: Consensus Building Workshop* What are the upstream policies needed to prevent NCD and related inequalities? (Group discussion and Plenary) Brainstorming possible NCD prevention policies; building a longlist; Shortlisting NCD prevention policies
**Workshop 4**	Discuss and learn about the upcoming NCD Prevention policy agenda at local and UK levels, and how best to influence it Build an understanding of the potential QUEST contribution to NCD prevention To develop a list of processes to ensure effective co-production, communication, and impact and outcomes for QUEST	*Activity 1: “Upstream” NCD Prevention policies and reducing inequalities:* Panel Discussion Brief, 5 minute presentations on: Current and future prevention policy priorities (1, 5, and 10 years) How policy agendas change Spotting windows of opportunity *Activity 2: Building a shared understanding of Co-Production:* Individual reflection, paired discussion, feedback in Plenary. Questions posed: 1. Co-production will be a priority for QUEST. How do you envisage this being done in a way that is productive, effective and practical? 2. What methods of communication to engage you as stakeholders would be the most convenient and efficient in the context of 5 year research programme? 3. What are the most effective approaches for knowledge brokering, knowledge exchange and translating evidence into action?

Abbreviation: NCD, non-communicable disease.

###  Data Collection and Analysis 

The design of the workshop programmes was theory-based using the Cairney/Oliver key co-production principles.^10,23^ In the context of this study, the co-production approach was used to enable researchers and stakeholders to work together to generate knowledge. The workshops were carefully planned using a “script approach.” We adapted elements of the Hovmand approach in order to structure and gently facilitate the workshop process.^24^The Hovmand approach uses small structured exercises with specific objectives and outputs and the extensive use of facilitation, discussions and analysis. An example script is provided in Supplementary file 1.

Following each workshop, 2 researchers (FLW, LH) undertook a thematic analysis^25^ of the meeting minutes, flip chart notes, consensus building workshop notes and group-work feedback. Familiarisation of the data was carried out, reading through all of the data and generating initial codes based upon the responses. These were then grouped into meaningful categories and further searched and reviewed for themes. The generated themes identified were used to inform discussion at subsequent workshops. This was an iterative process where our reports were then shared with participants to fill in any gaps.

 The research team reflected upon the process after each workshop. Reflection was also invited from participants as part of the general group discussion, particularly during the final workshop. Participants were also invited to send further thoughts after each workshop.

## Results

The participants’ prioritisation of potential NCD prevention interventions and policies proposed during workshops 2 and 3 is outlined in Tables 3 and 4. The numbers signify the amount of “votes” an intervention or policy received based upon the nominal group technique refinement of priorities. The emboldened text reflects the topics with the highest scores in each section. Overall, the big 6 upstream NCD drivers were identified: inequity, poor diet, tobacco, alcohol, inactivity and air pollution.

**Table 3:** Stakeholder Prioritisation of Potential NCD Prevention Interventions and Policies Proposed During Workshop 2 in Liverpool

**Broad Policy Area**	**Specific Intervention or Policy **	**MoSCoW Priority**			
		**Must Have **	**Should Have**	**Could Have**	**Would Like in Future**
Life skills	**School readiness and environment**	2*	**3**		
	**Strong leadership/action on mental health**	**1**	**2**	4	
	Reform of the education system		**1**	1	1
	Cooking skills for all adults and children			2	2
	More resources for early years literacy			1	1
Active design	**Active city planning**	**3**	**1**	1	
	**Affordable and efficient public transport**		**4**	3	
	Mile a day policy for all (settings)			1	1
	Mandatory 20MPH urban speed limit				2
Social policy	**Living wage**	**6**			
	**Affordable, warm, safe housing**	**3**		1	
	**Proportionate universalism**	**1**	**2**		
	Traffic light labelling on all food and drink				2
	Stop sport sponsorship by junk food			2	3
Food policy	**Reshape CAP agriculture **(Brexit opportunity)	**4**			
	Mandatory comprehensive alcohol labels			1	2
Regulation of risk factors	**Ban marketing of unhealthy products**		**1**	1	
	**Divestment in tobacco shares**		**1**		1
	Smoke free public places			2	
	Minimum tobacco purchase age raised to 21			1	3
	Phase out smoking in favour of e-cigarettes				
Fiscal policies	**A fairer tax system**	**4**		1	1
	**Tax unhealthy products**	**2**	**3**	1	
	Minimum unit pricing for alcohol		**2**	1	
	Junk food tax		**1**		
	Totally free childcare		**1**	2	
	Environment and active travel		**2**		2
	No new diesel cars sold after 2030				2
	Burden of proof in vehicle/cycling/pedestrian accidents				1

Abbreviation: NCD, non-communicable disease. * The numbers denote the number of participants who identified the intervention/policy as a priority.

**Table 4:** Stakeholder Prioritisation of Potential NCD Prevention Interventions and Policies Proposed During Workshop 3 in Glasgow

**Broad Policy Area**	**Specific Intervention or Policy **	**MoSCoW Priority**			
		**Must Have **	**Should Have**	**Could Have**	**Would Like in Future**
Commercial determinants of health	**Change public discourse to demand healthy environment**	3*			
	**A ction on promotions of unhealthy commodities**	**2**	**2**		
	Commercial/technological drivers of health/ill health/inequality	**1**		1	
	Tobacco pricing policies		**3**	1	
	Mandatory code of practice for advertising		**1**	1	
A life worth living and self worth	**What people need to have value in their life**	**5**	**5**	3	3
	**Upstream policy for improved p opulation mental h ealth**	**3**	**1**		
	Timelines for achieving public health goals ie, which generation is to benefit?	1	1		
Good places, better health	**Planning and infrastructure that creates healthy environments**	**4**			
	**Inequalities – physical, cognitive, and financial**	**1**	**1**	1	1
	**Public service reform, improved access to health and social care.**	**1**	**1**	1	
	**Health and health i nequalities in community -based planning**		**3**		
	Accessible public transport and active travel		**2**	2	
	Physical infrastructure design – streets, buildings, spaces		**1**	1	
	Expansion of free, high quality childcare			2	1
Income and employment	**Work for all**	**5**	**5**	3	4
	**Basic minimu m income and supporting w elfare system**	**4**			
	Reducing food poverty	**1**	**1**		
Regulation of harmful substances	Nationalisation of alcohol retail sales			2	1
	Legalisation of drugs			1	1

Abbreviation: NCD, non-communicable disease. * The numbers denote the number of participants who identified the intervention/policy as a priority.

Table 3 and Table 4 each outline just how favourably participants viewed the impact of broader fiscal policies such as fairer taxation, the introduction of a living wage, refining the Common Agricultural Policy and taxation on junk food. Whole group discussions focused upon inequalities as a major driver of ill health and the complexity of reducing health inequalities. For example, some public health interventions were perceived as increasing inequalities in health, there was a call for identifying ways that supported a reduction in inequalities rather than perpetuate them. Also, when trying to address inequalities, evidence was highlighted as an important factor, for example in relation to vehicle emissions, whether people in poorer communities are worst affected. However, such policy interventions are considered to be outside the scope of the traditional public health sphere, and would require support from elite actors in order to progress.

 The need for further training for policy-makers to enable them to appropriately review and interpret economic analyses such as return on investment was also seen as an important area of skills development.

###  Building Boundary-Spanning Solutions

 The divergence of the public health system across the 4 UK nations was seen as a potential challenge in terms of developing boundary-spanning solutions to these big problems. The increasing amount of decision-making power within the individual nations was thus felt to potentially undermine a UK-wide consensus. Understanding this territorial dynamic was considered crucially important for gaining political buy in.

 The ‘healthy environment’ was identified as an important emerging field, potentially including price, marketing restrictions and the built environment. Likewise getting progressively wider political buy in to prioritise the interlinked issues of sustainability, climate change, food production, diet, air quality and transport. The need to understand the interconnected nature, and role of diverse ‘policy actors’ across these areas was also considered vital.

###  The Policy Space

We identified a broad level of agreement regarding the importance of preventing NCDs and reducing inequalities, underpinned by a frustration relating to the evidence/policy gap.^10^

 Participants emphasised how the evidence/policy gap poses several challenges for reducing the incidence of NCDs including:

- A policy conundrum around generating robust evidence of the effectiveness of interventions when so few have been rigorously tried and tested.
- The need to advocate for bold policies, rather than incremental ones, (whilst recognising the political context where ‘bold’ policy may often not be favoured).
- Needing robust and timely evaluation to close this evidence/policy ‘gap.’

###  The Role of Evidence in NCD Prevention Policy

 While recognising the strong evidence for NCD risk factor epidemiology, the evidence underpinning the best NCD prevention policy interventions was considered to be patchier and more contested, particularly around the wider commercial and technological determinants of health. The over-reliance on evidence from randomised control trials was identified as a key issue, potentially biasing against “upstream” policy approaches while exaggerating the apparent importance of “downstream,” more easily trialed interventions. Reframing the debate to emphasize the key role of evidence from “real world” natural experiment and large cohort studies could enable policy-makers to better judge the usefulness of an intervention, particularly in issues where a randomized controlled trial would not be feasible.

 Participants also acknowledged that one must also focus on the implications for the wider economy.

###  The Role of Elite Actors

The contribution of ‘elite actors’ (ministers, public sector leaders) was seen as key to the success of specific policies,^15^ particularly the ‘buy in’ from leaders about a topic they were passionate about, and their overcoming opposition from vested interests. Participants therefore highlighted the importance of researchers and advocates engaging early with policy actors to develop relationships based on trust and shared understandings. These channels might then be used most effectively to generate meaningful change when a window of opportunity concerning a specific area of interest arose.

 The need for effective leadership at local and national government levels was also emphasized, in order to inspire and enable other actors to contribute toward the effectiveness and success of interventions and policies. Major change could occur locally where there was good leadership – as exemplified by tobacco control successes.

The role of industry actors was also seen as being crucial, given their level of influence and track record of effectively undermining attempts to implement national public health legislation, via marketing, lobbying and denialism tactics.^26^ The example of Public Health England partnering with the alcohol industry funded Drinkaware was identified as a topical concern in Summer 2018, particularly given the industry simultaneously investing £9m in the campaign against minimum unit pricing for alcohol.^27^

###  Optimising Co-Production and Communication 

 Workshop 4 focused particularly on effective methods of co-production, evidence generation, communication and dissemination, and the further development of the QUEST research consortium. The need to identify evidence gaps, harness existing alliances and prioritise a small number of key issues was seen as a positive way forward. It was agreed that the principles of co-production meant maintaining positive relationships, particularly in areas of disagreement due to a lack of scientific evidence (such as electronic cigarettes). This helped to identify and further develop areas of consensus, even in the context of contested issues or evidence, and also lay the groundwork for effective and rapid action when new insights and opportunities emerged, (recognising the continuously evolving nature of social, political, and research systems).

###  Maximising the Value of the QUEST Research Consortium

 Workshop participants identified the need to actively build a sense of common purpose across the diverse consortium of stakeholders. A comprehensive communications strategy was thus considered essential, targeting professionals, policy-makers, politicians and the public.

 Maintaining momentum over a 5-year research programme was considered potentially challenging due to issues of the non-continuity of key individuals in specific roles within their stakeholder organisations. Regular face-to-face contact with a core group of stakeholders (senior decision-makers from a diverse group of organisations relevant to public health policy) was identified as a way to mitigate this, particularly through regular meetings, events and annual conferences.

## Discussion

We identified a broad level of stakeholder agreement regarding the importance of preventing premature NCDs and reducing inequalities, underpinned by frustrations regarding the evidence/policy gap. While recognising the strong evidence for NCD risk factor epidemiology, the evidence underpinning the best NCD prevention policy interventions was patchier and more contested around the wider determinants of health. Although a wide array of societal factors are known to influence health, the evidence regarding the specific interventions to best address them is sparse, particularly at local and regional levels.^28,29^

A comprehensive communications strategy was considered essential, targeting professionals, public, policy-makers and politicians. The contribution of ‘elite actors’ (ministers, public sector leaders) was therefore seen as key to the success of policies, including emotional ‘buy in’ from leaders about a specific topic, and robustly negating opposition from commercial vested interests.^10^

There was also broad agreement of the need to prioritise a small number of topics in order to maximise effectiveness and national impact. This was as much a consequence of the time-limits and resource-constraints of public health research projects. Likewise, the recognition that policy action is strongly structured by the policy ‘agenda’: at any one moment, policy actors only have the time and cognitive space to pay attention to a very short list of salient issues.^30^

There was a clear need to define how QUEST researchers engaged specifically with the devolved nations given the progressively fragmented nature of the national legislative framework. Whilst many interventions might work on a UK wide footprint, others would have greater buy in from regional political leaders if targeted specifically at the devolved nations’ specific concerns.^31^

Several participants cited healthy diet, air quality, public transport and climate change as key tenets of a good public health system. The wider political and social acceptance of the climate emergency has thrust these public health pillars increasingly into the spotlight.^32^ It is thus imperative that the public health community is able to respond comprehensively and cohesively, particularly now that the wider policy system is better aligned.^33,34^

These findings complement and strengthen existing knowledge. Breda et al^35^ call for NCD prevention interventions and policies to include “multi-stakeholders” beyond the traditional health sector at all stages of consideration and development, taking into account possible competing interests, and having evidence informed and context relevant implementation. Isaranuwatchai et al^36^ discuss the importance of local context in making decisions about implementing interventions for preventing NCDs, in terms of assessing “best buys, wasted buys, and contestable buys” particularly in relation to equitability and context.

###  Implications for Policy, Public Health, and Future Research

The early and ongoing engagement of diverse stakeholders in co-production provided valuable outputs in our project, as in earlier ones, helpfully informing context, relevance and reality checks around potentially feasible prevention strategies.^16^ Ongoing partnership working with stakeholders likewise remains essential for the expert interpretation of emerging findings and optimisation of policy dissemination and implementation.^37,38^

However, achieving this in practice requires an awareness of the diversity of organisations and government departments involved in developing public health policy. Furthermore, maintaining such awareness can be challenging at a local or regional level.^18,38^

The “wicked problem” of NCD prevention in a complex political and public health environment therefore requires sustained input at national, regional and local levels.^39^ Limited time and resources, as well as competing priorities, highlights the need for greater co-operation and co-production amongst stakeholders across the UK public health community. Research consortia like QUEST therefore potentially offer a unique opportunity to provide a local and national perspective and facilitate knowledge exchange.

Future research would benefit from explicitly acknowledging this complexity.^8^By using a systems approach, we might better synthesise different forms of information, integrating stakeholder perspectives and best evidence into our innovative, quantitative policy models. Further testing of the outcomes of such systems design would produce new knowledge and we could then better identify the most equitable prevention strategies, and then help translate this compelling evidence into policy practice.^40^

Further examination of the co-production process^15,16^ could also be valuable, particularly how it might bring senior stakeholders together to build consensus, develop policy options and thus ensure research is relevant and timely to the needs of policy and decision-makers.

###  Strengths

We describe a carefully developed process and qualitative inquiry using co-production methodologies to develop an overview of NCD prevention policies within a complex environment. This approach was built on a solid theoretical foundation, combining insights from the literature on the co-production of policy knowledge with those from the policy literature.^10,19,31,33,34^ The former underpinned the establishment of fruitful dialogue between researchers and various policy actors, all of whom were not considered to be part of two distinct and separate ‘communities’ but rather whose different perspectives and inputs were relevant, albeit in different ways, to generating actionable policy-relevant knowledge.^31^ The latter underscored the importance of fostering relationships between policy actors, including researchers, to not only translate various types of knowledge but also to identify and create opportunities for action and establish an environment into which new evidence on how to reduce the prevalence of NCDs could quickly be taken up and acted on. As such, stakeholders were drawn from a diverse array of local, regional and national organisations, reflecting the territorial and sector complexity into which any successful NCD-reduction policies will have to fit. Furthermore, we included very senior public health leaders from England and Scotland, providing crucial context in these increasingly divergent public health systems.

###  Limitations

 Such research inevitably has limitations. Firstly, devolved public health systems are complex and different – what works in one place might not necessarily work in another for a wide variety of political, cultural and societal factors. Further research would therefore benefit from replication involving representatives from Wales, Northern Ireland and a wider range of local authorities. Secondly, our final workshop focused particularly on co-production. However, it was clear that the very interesting and useful discussions required more time in order to fully realise the opportunities that this exciting approach potentially has to offer. Thirdly, this study was limited to researchers and elite players, but ‘real-world’ evidence relating to equitable solutions may benefit from the intended beneficiaries’ involvement. There would be value in developing a second stage with inclusion of the public. Finally, our workshops offer a snapshot of a specific point in time. Discussions around climate change, food production, e-cigarettes etc have all progressed further since then. However, the principle findings remain valid.

## Conclusion

 NCD drivers like poor diet, smoking and alcohol reflect complex adaptive systems. Strategies to prevent premature NCDs therefore potentially represent “wicked” problems. However, the early and ongoing engagement of diverse stakeholders in co-production could well be valuable, potentially providing context, relevance and reality checks regarding feasible strategies. Continued joint working with these partners could then optimise the co-production, dissemination and implementation of the potentially most impactful policy solutions.

## Acknowledgements

 We would like to thank all the stakeholders who participated in the workshops and provided us with their insight and expertise. The workshops were funded by UK Prevention Research Partnerships (award UKPRP_CO1_105).

## Ethical issues

 All participants were informed in advance of the purpose and format of the workshops. Verbal consent for group feedback and discussion to be recorded in note form was obtained. No identifiable individual comments or expressed views were used.

## Competing interests

 RM is employed by Public Health Wales, and holds an Honorary Research Fellowship with the University of Liverpool. LH, MO, FLW, and SC are employed by the University of Liverpool. ESD is employed by the University of Stirling.

## Authors’ contributions

 FLW: conceptualization, data curation, funding acquisition, investigation, methodology, project administration, resources, writing – original draft, review and editing. RM: Formal analysis, roles/writing – original draft, writing – review and editing. LH: Data curation, investigation, project administration, writing – review and editing. ESD: Writing – review and editing, methodology: conceptualization, data curation, funding acquisition, investigation, project administration, resources, writing – review and editing. SC: Conceptualization, data curation, funding acquisition, investigation, methodology, project administration, resources, writing – review and editing.

## Funding

 This work was funded in part by a consortium development grant from the MRC UK Prevention Research Partnership (UKPRP_CO1_105).

## Supplementary files

Supplementary file 1. Detailed Plan for QUEST Workshop 3.
