# Supplementary material for: The QUEST for Effective and Equitable Policies to Prevent Non-communicable Diseases: Co-Production Lessons From Stakeholder Workshops
Source: Int J Health Policy Manag. 2020 Jun 28;10(10):638–46. doi: 10.34172/ijhpm.2020.99 (PMC9278532; doi:10.34172/ijhpm.2020.99)
Supplement: Supplementary file 1 — Detailed Plan for QUEST Workshop 3. [file ijhpm-10-638-s001.pdf]

Supplementary file 1. Detailed Plan for QUEST Workshop 3

13.30 – 15.30, TUESDAY 4th September, Glasgow City Centre Hotel

Materials List

- Digital projector and laptop
- Name Tags
- Flip chart markers black (1 for each participant plus extra for facilitators n = 25)
- A5 white paper (120 sheets)
- A5 pink paper (30 sheets)
- A5 blue or green paper (30 sheets)
- 3M Blue Painters tape (2 rolls)
- 4 different colour star/dot stickers for 20 participants (ie, 25 each colour = 100 stickers in total)
- Name cards for tables
- Flip chart paper

FLW: ***Workshop Convener/Closer:*** Primary responsibility for starting the session, introducing participants to the exercise, making sure that participants understand the purpose of the exercise within the context of their organization or community, and introducing the facilitators. Closer has primary responsibility for bringing the session to close and thanking participants for their time.

MG-C (with assistance from FLW and LH as required): ***Wall Builder:*** The primary responsibility of the wall builder is to organize products from an exercise into thematic clusters, as well as to explain the clusters to the participants in order to elicit their feedback.

FB,LH, FLW AND MG-C: ***Note Takers:*** Primary responsibility for taking notes about what is said in the workshop.

LH: ***Time Keeper:*** Primary responsibility for notifying the facilitation team when time is short. It is overall very important to start and end on time as much as possible.

MOF AND JP-S: ***Reflectors:*** Primary responsibility for helping the group reflect on what they have done and recognize the issues/insights that have been developed during the workshop. This role requires someone who can speak to the relevance of the activities and insights to a larger substantive context.

Consensus Workshop Process – Script Summary

**Step**	**Purpose**	**Timing**	**Roles**
Focus Question	***What are the upstream policies needed to prevent NCD and related inequalities?***		

Rational Aim	To develop a list of priority upstream policies in preventing NCD and related inequalities	N/A	
Experiential Aim	For the participants to be happy with, engaged by and enthused by QUEST	N/A	
CONTEXT<br><br>**13.30 – 13.45**	• Aim of the workshop<br>• Clarify focus question<br>• Outline the process<br>• Brief, focussed conversation to initiate thinking about the question at hand	15 minutes	**FLW** introduces the activity, stating the focus question, aim, process and expected time. Beginning with a focused conversation related to the focus question to get people thinking about their own experiences and knowledge (Before we start the main activity we would like you to think about your own experience and knowledge in terms of policies to prevent NCD and related inequalities – then ask people to share with group).<br><br>**LH** to keep time
BRAINSTORM<br><br>**13.45 – 14.05**	• Instructions<br>• Individually *(as many as they can think of "brain dump")*<br>• In groups, Prioritise 9 policies to share<br>• Three clearest ideas sent to the front to go on the wall – placed there randomly<br>• Clarification of anything that is not clear	20 minutes (Total)<br><br>(5 minutes)<br><br>(10 minutes)<br><br>(5 minutes)	**FLW** asks the group to individually brainstorm upstream policies. Then within their group to priorities 9 policies to share.<br><br>**MG-C & LH** collect first 3 cards from groups and place on wall randomly.<br><br>**MG-C/FLW** ask group if any cards require clarification and/or any cards we are not clear about.<br><br>**LH** to keep time
CLUSTER<br><br>**14.05 – 14.25**	• Find natural clusters in the cards already on the wall, leaving those that don't fit as they are<br>• Ask for a second set of 3 cards that are different<br>• Develop clusters of ideas – label these<br>• Pass up remaining cards and anything else that is burning but didn't make it to the list – labelled with a cluster symbol	20 minutes (Total)<br><br>(10 minutes)<br><br>(10 minutes)	**MG-C with FLW** ask the group to find natural clusters<br><br>**MG-C & LH** collect next 3 (different) cards from groups and place on wall randomly.<br><br>**MG-C** develops cluster of ideas.<br><br>**MG-C & LH** collect remaining (different) cards from groups

			and other cards that did not make the group list, but individually seen as important place on wall randomly.<br>**MG-C** develops cluster of ideas.<br>**FLW** will put cluster symbols against the emerging groups.<br>**LH** to keep time
NAME<br>**14.25 – 14.45**	• Talk through and refine the clusters<br>• Give each cluster a name that answers the focus question	20 minutes (total)<br>(5 minutes)<br>(15 minutes)	**MG-C & FLW** talk through the clusters asking the group to refine the identified clusters.<br>**MG-C & FLW** ask group to provide titles/names for the clusters relating to types of policy they relate to.<br>**FLW** writes titles on cards and with **MG-C** places them on the wall.<br>**LH** to keep time
RESOLVE<br>**14.45 – 14.55**	• Read through all the title cards<br>• Discuss the significance of the consensus	10 minutes	**MOF/JP-S** reflect upon the title groups identified and invite the stakeholder group for comments<br>**FB; LH; FLW; MG-C** to take notes of this 10 minute discussion<br>**LH** to keep time<br>**FLW** to close session and say that we will be returning to the wall after the break to shortlist the identified policies
**COMFORT BREAK**<br>**14.55 – 15.00**			
**MoSCoW**<br>**15.00 – 15.30**	To create a shortlist of the NCD prevention policies identified	30 minutes (total)	**MG-C** provides presentation of the MoSCoW approach.<br>Stakeholders have opportunity for questions

		10 mins presentation<br><br>5 minutes for questions<br><br>5 minutes for placing dots<br><br>5 minutes for Maria to summarise	**MG-C** asks stakeholders to place coloured dots against the policies which are: Pink – Must Have; Blue – Should Have; Red – Could Have; Yellow – Would Have in the Future<br><br>**FLW/LH** to have the coloured dots ready on each table for stakeholders to use.<br><br>**MG-C** summarises the dot voting of the policies<br><br>**LH** to keep time
